# Imaging analysis of the biological parameters of the lens in patients with cortical age-related cataracts using ultrasound biomicroscopy

**DOI:** 10.1186/s12886-023-03227-2

**Published:** 2023-11-22

**Authors:** Zhiying Yu, Na Li, Fenglei Wang, Jing Fu, Shasha Xue, Ling Wang

**Affiliations:** 1 Department of Ophthalmology, The Affiliated Hospital of Qingdao University, Qingdao, China; 2 The Affiliated Hospital of Qingdao University, Qingdao, China

**Keywords:** Cortical age-related cataract, Ultrasound biomicroscopy, Lens vault, Iris-lens angle, Iris-lens contact distance, Lens position

## Abstract

**Background:**

The spatial position of the lens in patients with cortical age-related cataract (CARC) is unclear. We investigated a basis for the assessment of visual quality after cataract surgery by analysing the ultrasound biomicroscopic characteristics of the biological parameters of the lens in patients with CARC.

**Methods:**

In this retrospective study, 119 patients (50 males and 69 females, totalling 238 eyes) with CARC who underwent simple cataract surgery were selected. The lens thickness (LT), axial length (AL), anterior chamber depth (ACD), lens vault (LV), trabecular-iris angle (TIA), iris-lens angle (ILA), iris-lens contact distance (ILCD) were measured by A-scan ultrasound and ultrasound biomicroscopy. The corresponding lens position (LP) and relative lens position (RLP) were calculated.

**Results:**

LP was greater in men than in women (*P* < 0.05), LV was smaller in men than in women (*P* = 0.002), ILA and ILCD were not statistically significant (*P* = 0.072 and P = 0.854, respectively). There were significant differences in TIA, ILA, and ILCD in the four quadrants (all *P* < 0.05), with a trend in the distribution of TIA: superior < inferior < nasal < temporal, ILA: nasal < inferior < temporal < superior, and ILCD: superior < temporal < inferior < nasal.

**Conclusions:**

The lens protrudes more obviously in females than in males and the lens tilts to a certain extent with the increase of age and tends to be more upward and temporal in the supine position. Therefore, trends in lens-related parameters in patients with CARC should be taken seriously.

## Introduction

Cataract is one of the important causes of visual impairment and reversible blindness worldwide [1]. With the aging of the global population, the prevalence of age-related cataracts will continue to increase, and cataract surgery is the main solution [2]. However, with increasing age, the structure and position of the lens play an important role in the narrowing of the anterior chamber angle, and more attention is being paid to the role of lens-related parameters in the narrowing of the anterior chamber angle [3–5]. In addition, changes in lens-related parameters, especially whether changes in lens position will affect the safety of cataract surgery and postoperative visual quality, have attracted the attention of ophthalmologists [6]. What is the change regularity of lens-related parameters such as LV, ILA, ILCD, LP and RLP in the age-related cortical cataract population? What is the change trend of lens-related parameters in different quadrants? So far, there are few related studies.

Ultrasound biomicroscopy (UBM) is an important tool for examining the anterior segment of the eye [7], which can clearly display images of the anterior and part of the middle segment of the eye and measuring the values of various biological parameters [8]. It can accurately display the tissue structure of the anterior segment, not be affected by the refractive media, and can quantitatively measure relevant parameters [9]. In this study, we assessed 119 patients (238 eyes) with CARC using UBM, and analysed the characteristics of lens-related parameters in different genders and different eyes, as well as the distribution characteristics of lens-related parameters in four different quadrants. We believe this can provide some important information about the role of lens-related parameters in the process of anterior chamber angle narrowing. In addition, the assessment of lens deviation based on preoperative measurements of lens-related parameters can guide the clinical selection and adjustment of intraocular lens (IOL ) parameters, so as to obtain satisfactory surgical results.

## Patients and methods

### Study design and patients

In this retrospective study, 119 patients (50 males and 69 females, totalling 238 eyes) with CARC who underwent simple cataract surgery at the Ophthalmology Department of the Affiliated Hospital of Qingdao University from September 2020 to June 2022 were selected. Their ages ranged from 41 to 86 years (mean, 66.410 ± 9.395 years)(Table 1). The inclusion criteria were: (1) Patients with CARC in both eyes (and both in the intumescent or mature stage according to the image staging on UBM [10]) and at least one eye requiring surgical treatment; and (2) Age ≥ 40 years. The exclusion criteria were: (1) Patients with a history of ocular surgery or trauma; (2) Patients with mobility problems who could not lie on their backs; (3) Patients with poor cooperation and anaesthetic allergy; (4) Patients with high myopia and an axial length > 27 mm; and 4) Patients with high intraocular pressure (> 21 mmHg). This study met the requirements of the Ethics Committee of the Affiliated Hospital of Qingdao University and was conducted in accordance with the Declaration of Helsinki.

**Table 1:** General information on the patients

Varible	Male(n = 50)	Female(n = 69)	Total(n = 119)
Mean age, years	64.680 ± 9.377	67.670 ± 9.273	66.410 ± 9.395
Age range, years	45–84	41–86	41–86
Number/percentage	50(42.02%)	69(57.98%)	119(100%)
F and *P*	0.0200.888		-
t and *P*	-1.7260.087		-

Note: The age parameters of the patients of different genders were tested using an independent samples t-test. The variance of age parameters of cataract patients of different genders was consistent (*P* = 0.888), and the difference was not statistically significant (*P* = 0.087) based on the t-test, indicating that there was no significant difference in age among cataract patients of different genders we selected

### Research methods

Each patient underwent slit-lamp microscopy, visual acuity, intraocular pressure, and computerized optometry, and A-ultrasound and UBM examinations were performed by the same experienced technicians.

Ophthalmic A-ultrasound: (Quantel Medical, France), patients were placed in the supine position and, were asked to look at the finger directly above them, after the application of a topical anaesthetic. Then the LT and AL of each patient were measured separately, with each parameter measured ten times and averaged.

UBM: (3200 L, Tianjin Suowei; probe frequency, 50 MHz), the examination was performed after dripping topical anesthetic under natural light, and the patients were placed in the supine position. Scan the images of the anterior chamber and the angle images of the superior, inferior, nasal and temporal (corresponding to 12, 6, 3, and 9 o’clock, respectively, in the case of the right eye). Save the images with better quality, and ACD and LV were measured using the system’s software. At the same time TIA, ILA and ILCD in four directions of the superior, inferior, nasal and temporal (for example, 12 o’clock, 6 o’clock, 3 o’clock, and 9 o’clock for the right eye) were measured. The corresponding LP and RLP were calculated using the equations: LP = ACD + 1/2LT and RLP = LP/AL [11, 12].

### Measurement parameters

**Fig. 1:**
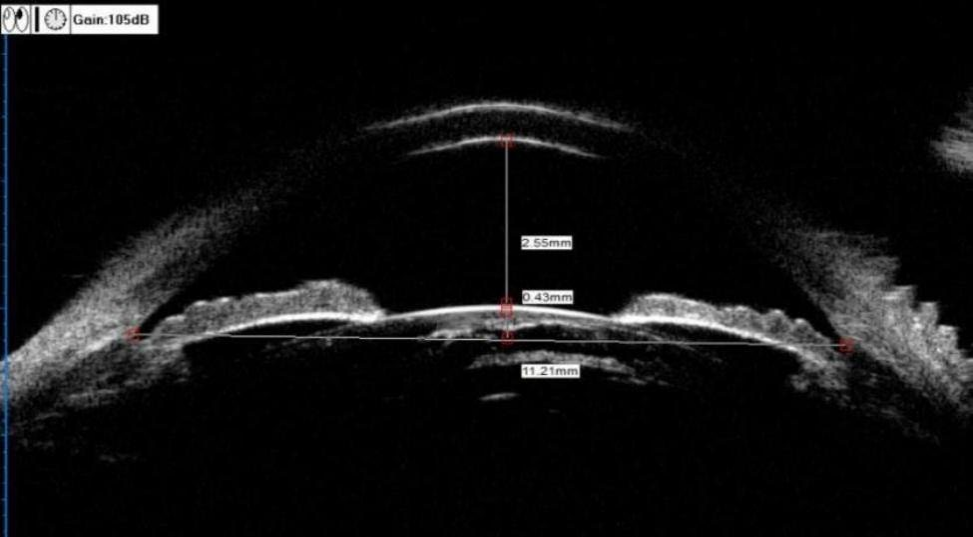
UBM measurement of ACD and LV ACD = 2.55 mm; LV = 0.43 mm

ACD (mm): was the vertical distance from the inner surface of the central cornea to the anterior surface of the lens, as shown in Fig. 1.

LV (mm) [4]: was measured as the perpendicular distance from the anterior pole of the lens to the horizontal line between the scleral spurs, as shown in Fig. 1.

**Fig. 2:**
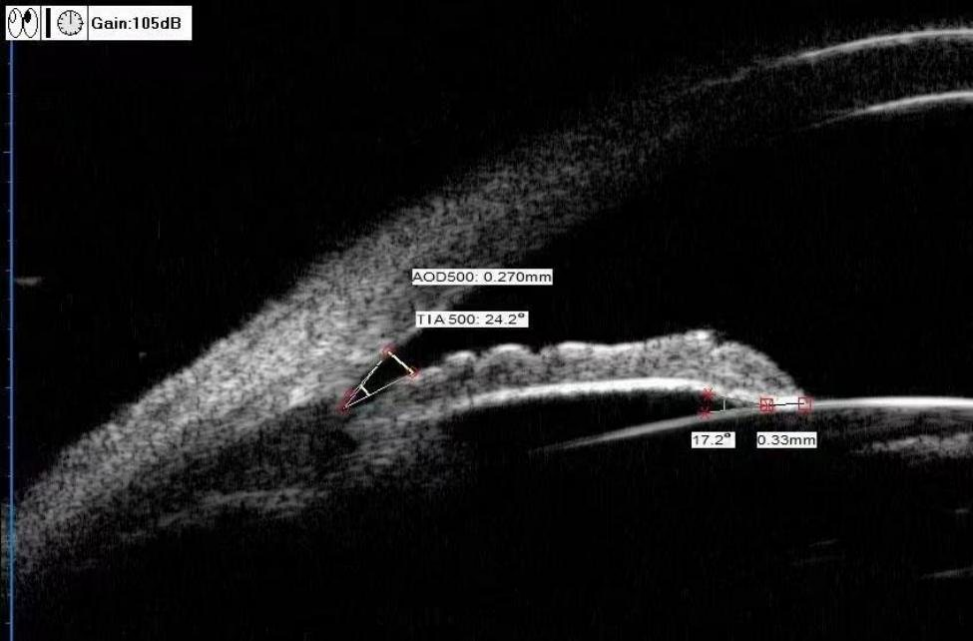
UBM measurement of TIA, ILA and ILCD. TIA = 24.2°; ILA = 17.2°; ILCD = 0.33 mm

TIA (°) [13]: This value was consistent with the anterior chamber angle of 500 μm (anterior chamber angle at 500 μm from the scleral spur, ACA500). The specific measurement method was to make a triangle with angle opening distance (AOD500) as the base and the recess at the iris root as the vertex. The angle of the vertex was the TIA, as shown in Fig. 2.

ILA (°) [13]: The specific measurement method was to take the contact point between the posterior iris surface and anterior lens surface as the vertex. Two sides along this vertex were tangent lines of the posterior iris surface and anterior lens surface. The angle formed was the ILA (Fig. 2).

ILCD (mm) [13]: was the line between the contact points of the anterior and posterior iris surfaces and the anterior lens surface (Fig. 2).

### Statistical methods

SPSS 19.0 software was used to analyse the data. If the parameters were normally distributed, the results were expressed as mean ± standard deviation. *P* < 0.05 indicated a statistically significant difference, and *P* < 0.01 indicated a extremely distinct difference If the parameters were not normally distributed, quartiles were used for the statistics. Measurements were analysed using the t-test for comparisons between two groups and the non-parametric Kruskal-Wallis test for comparisons among four groups.

## Results

### Effect of gender on the lens-related parameters

As shown in Table 2, the mean TIA was 19.544 ± 8.623° for male patients with cataracts, which was greater than that for females (15.669 ± 9.475°), with a significant difference (*P* = 0.001). The mean ILA for males (13.855 ± 4.069°) was also greater than that for females (12.910 ± 3.923°). Moreover, the mean ILCD for males (0.588 ± 0.191 mm) was less than that for females (0.593 ± 0.209 mm). None of the differences were significant (*P* = 0.072 and *P* = 0.854). The LP for males (4.751 ± 0.305 mm) was greater than that for females (4.590 ± 0.349 mm), with a significant difference (*P* < 0.001). The RLP for males (0.201 ± 0.015 mm) was greater than that for females (0.199 ± 0.015 mm), with no significant difference (*P* = 0.208). Furthermore, the LV for males (0.308 ± 0.257 mm) was smaller than that for females (0.417 ± 0.260 mm), with a significant difference (*P* = 0.002).

**Table 2:** Comparison of the lens-related parameters in different genders

Parameters	Male(n = 100)	Female(n = 138)	Levene test		T-test	
	Mean ± SD	Mean ± SD	F	*P*	t	*P*
Mean TIA(°)	19.544 ± 8.623	15.669 ± 9.475	1.911	0.168	3.233	0.001*
Mean ILA(°)	13.855 ± 4.069	12.910 ± 3.923	1.022	0.313	1.806	0.072
Mean ILCD(mm)	0.588 ± 0.191	0.593 ± 0.209	0.642	0.424	-0.184	0.854
LV(mm)	0.308 ± 0.257	0.417 ± 0.260	0.772	0.381	-3.198	0.002*
LP(mm)	4.751 ± 0.305	4.590 ± 0.349	1.979	0.161	3.702	<0.001*
RLP(mm)	0.201 ± 0.015	0.199 ± 0.015	0.939	0.334	1.262	0.208

*:Statistically significant at *p* < 0.05

### Effects of different eyes on lens-related parameters

As shown in Table 3, the mean values of the ILCD (0.593 ± 0.208 mm), LV (0.373 ± 0.258 mm), LP (4.681 ± 0.354 mm), and RLP (0.201 ± 0.015 mm) in the right eye were greater than those of the ILCD (0.588 ± 0.195 mm), LV (0.369 ± 0.271 mm), LP (4.634 ± 0.325 mm), and RLP (0.199 ± 0.014 mm) in the left eye, but the differences were not significant,. Moreover, the mean values of the ILA (13.145 ± 3.945°) and TIA (16.801 ± 9.144°) in the right eye were smaller than those of the ILA (13.469 ± 4.072°) and TIA (17.794 ± 9.481°) in the left eye, without significant differences.

**Table 3:** Comparison of the lens-related parameters in different eyes

Parameters	Right Eye(n = 119)	Left Eye(n = 119)	T-test	
	Mean ± SD	Mean ± SD	t	*P*
Mean TIA(°)	16.801 ± 9.144	17.794 ± 9.481	-1.639	0.104
Mean ILA(°)	13.145 ± 3.945	13.469 ± 4.072	-1.251	0.213
Mean ILCD(mm)	0.593 ± 0.208	0.588 ± 0.195	0.446	0.656
LV(mm)	0.373 ± 0.258	0.369 ± 0.271	0.243	0.809
LP(mm)	4.681 ± 0.354	4.634 ± 0.325	1.723	0.087
RLP(mm)	0.201 ± 0.015	0.199 ± 0.014	1.658	0.100

### Comparison of the lens-related parameters between different quadrants

Table 4 shows the distribution of the mean TIA in all four quadrants. The trend was as follows: superior < inferior < nasal < temporal, with significant differences between the four quadrants.

**Table 4:** Comparison of the TIA between different quadrants

	N	Superior	Inferior	Nasal	Temporal
		P50(P25, P75)	P50(P25, P75)	P50(P25, P75)	P50(P25, P75)
TIA	238	9.500(4.600, 18.075)	13.150(6.875, 21.125)	20.850(10.275, 30.325)	21.350(14.100, 29.325)
H and *P*		137.032		<0.001*	

*:Statistically significant at *p* < 0.05

Table 5 shows the distribution of the mean ILA in all four quadrants. The trend was as follows: nasal < inferior < temporal < superior, with significant differences between the four quadrants.

**Table 5:** Comparison of the ILA between different quadrants

	N	Superior	Inferior	Nasal	Temporal
		P50(P25, P75)	P50(P25, P75)	P50(P25, P75)	P50(P25, P75)
ILA	238	13.600(10.975,16.525)	12.800(9.500,16.325)	11.200(8.775,14.500)	13.250(10.000,16.500)
H and *P*		31.729		<0.001*	

*:Statistically significant at *p* < 0.05

Table 6 shows the distribution of the mean ILCD in all four quadrants. The trend was as follows: superior < temporal < inferior < nasal, with significant differences between the four quadrants.

## Discussion

Cortical cataract is the most common type of age-related cataract [14]. Especially during the intumescent stage, when the opacity of the lens increases with age, the cortex absorbs water and the lens volume increases, causing the anterior chamber to become shallower [15, 16] and possibly triggering an acute attack of glaucoma in patients with narrow anterior chamber. In the process of anterior chamber angle narrowing, the changes and roles of lens-related parameters have attracted more and more attention from scholars, with LV, LP, and RLP being more frequently studied [17–19]. In our previous study, we found that the ILA and ILCD were characteristically present in eyes with acute lens-induced angle closure [20]. However, there are no studies on whether the ILA and ILCD are different in each quadrant of patients with CARC. On the other hand, whether this change in lens-related parameters that occur with age affects the improvement of visual quality after cataract surgery remains debatable.

Most studies regarding the lens have been performed using anterior segment optical coherence tomography (AS-OCT), and relatively few using UBM. AS-OCT is widely used in clinical practice due to its non-contact, non-invasive, and maneuverable. It has the advantage of being safe, unaffected by the degree of corneal clouding and clearly showing some anterior chamber angle structures, such as the scleral spur [21]. However, the posterior iris structures are poorly displayed with AS-OCT due to the influence of the iris pigment epithelium and refractive media. UBM is a high-resolution ultrasound technique that provides a detailed assessment of the anterior segment of the eye [22], which is independent of light and refractive media, and has the advantages of reproducibility, objectivity, and reliability [23]. However, it is relatively complex to perform and requires a water bath. In the previous study on the consistency of these two measurement methods for ILA and ILCD, we found that the consistency of the two measurement methods in the superior, inferior, nasal, and temporal directions is good for ILCD, but the consistency for ILA measurement is poor, which may be related to poor imaging of the posterior iris tissue by AS-OCT [24]. This means that although AS-OCT is more convenient, the use of UBM is more dominant for measuring the lens-related parameter, such as ILA. Cataract surgery is generally performed in the supine position, which is the same as the examination position for UBM, the results of UBM are closer to the intraoperative state of the anterior segment. Therefore, we chose UBM to study and analyse the characteristics of the lens-related parameters in different genders, different eyes, and different quadrants of CARC.

LV, a new risk factor [25], is closely associated with anterior chamber angle closure [4]. Furthermore, a greater forward protrusion of the lens may result in a narrower anterior chamber angle which is more likely to induce progressive anterior chamber angle closure [26]. The larger the LV, the more the iris pushes anteriorly, increasing the contact between the iris and the lens, subsequently increasing the ILCD, thereby causing pupillary block [27]. A study by Tan et al. [28] showed that the LV increased significantly with age, its value in female patients was greater than that in male patients, and it was associated with narrow anterior chamber angle. Our study showed that the mean TIA in males was greater than that in females, while the mean LV in males was less than that in females. Moreover, the mean ILA in males was greater than that in females, while the mean ILCD in males was less than that in females. However, none of the differences were significant. The LV represents the extent to which the lens protrudes into the anterior chamber [3]. Further, as shown in our study, the larger the LV in females, the more the lens protrudes forward and pushes the iris, thus corresponding to a larger ILCD and a smaller ILA.

Both LP and RLP reflect the influence of the anatomical position of the lens on the anterior chamber structure. Lim et al. [29] assessed the eyes with the acute attacks and the fellow eyes in patients with PACG and compared the ACD, LP, RLP, and other related variables. They found that the LP was more anterior in the eyes with acute attacks than in the fellow eye in those patients, while there was no significantly different for RLP between the two groups. However, Nongpiur et al. [3] used AS-OCT to compare PACG patients with normal subjects, and found that LP and RLP had no correlation with the occurrence of PACG. Our study showed that the mean LP was larger in males with CARC than in females, with a statistically significant difference. Contrastingly, the difference in RLP between the male and female patients was not significant. A study by Pakuliene et al. [30] concluded that the LP could be used to predict narrow angles, and it was greater in males with cataracts than in females. Moreover, the TIA was also greater in males than in females, which is consistent with our results. The LP and RLP are affected by the ACD and LT, the correlation between these two parameters and anterior chamber angle closure needs further investigation, but women have a more anteriorly positioned lens, crowded anterior structures in the eye, and narrower anterior chamber angles, which are more likely to induce acute glaucoma attacks.

The preoperative position and inclination of the lens will affect its postoperative visual acuity and visual quality. Therefore, many scholars have conducted studies on this. Chen et al. [31] studied the characteristics of lens inclination and eccentricity in 1097 patients proposed for cataract surgery using CASIA2. They found that the average inclination of the natural lens to the infratemporal direction is 5.16°, and the average eccentricity to the temporal side is 0.22 mm. It was concluded that a certain degree of inclination and eccentricity existed in the preoperative lens of patients with cataracts. Li et al. [32] studied 230 patients aged 7–90 years using CASIA2 and found that the lens tilted to the infratemporal, with a mean inclination of 4.3 ± 1.5° and an eccentricity of 0.17 ± 0.12 mm to the super-temporal. In contrast, Wang et al. [33] retrospectively studied the lens and IOL of 333 patients (mean age, 70 ± 8 years) who underwent cataract surgery using the IOL Master 700. They found that both the lens and IOL tilted to the nasal side with a mean amplitude of 3.7 ± 1.1° and 4.9 ± 1.8°, respectively. Kimura et al. [34] assessed 100 eyes (the lens of 41 eyes and the IOL of 59 eyes) in 49 patients using CASIA2. They found that in the non-dilated state, the lens tilted at an average angle of 5.15° to the infratemporal, and the IOL tilted at an average angle of 4.31° to the infratemporal. On the other hand, in the dilated state, the lens tilted at an average angle of 5.25° to the infratemporal, and the IOL tilted at an average angle of 4.65° to the infratemporal. Our measurements showed that the distribution of the ILA tended to be nasal < inferior < temporal < superior. The H-test showed that there were significant differences in anterior chamber angles of the four quadrants, while the mean distribution trend of ILCD in the four quadrants is just the opposite: superior < temporal < inferior < nasal, with significant differences between the four quadrants. Combining the results of ILA and ILCD, the lens shifted upward and temporally. Differences in these measurement results may be related to differences in the study population, measurement positions, measurement methods, and instruments used. Cui et al. [35] used UBM to observe the preoperative lens zonulas in 50 eyes of 50 patients with cataracts combined with PACG. They found that the zonulas was abnormal in 29 eyes (58%), of which nine eyes (18%) were disconnected, and 20 (40%) were loose. The zonulas of the lens may become loose with age and degeneration. Therefore, we hypothesise that during a seating examination, such as an AS-OCT, the lens is more likely to be deflected downwards due to gravity. During the operation position (supine position) examination, the lens of some patients will have a slight upward deviation when the head is slightly tilted back.

**Table 6:** Comparison of the ILCD between different quadrants

	N	Superior	Inferior	Nasal	Temporal
		P50(P25, P75)	P50(P25, P75)	P50(P25, P75)	P50(P25, P75)
ILCD	238	0.530(0.400, 0.693)	0.570(0.440, 0.720)	0.575(0.450, 0.773)	0.540(0.400, 0.700)
H and *P*		8.745		0.033*	

*:Statistically significant at *p* < 0.05

## Conclusion

Overall, based on the imaging data of patients with CARC from UBM, we observed change patterns of the anterior chamber angle and lens parameters based on different genders, eyes, and quadrants. This can guide us to pay attention to the role of lens-related parameters in the narrowing of the anterior chamber angle in patients (especially female patients) with CARC. In addition, the assessment of lens deviation based on preoperative measurements of lens-related parameters can guide the clinical selection and adjustment of IOL parameters rationally, leading to satisfactory surgical outcomes. However, future studies on how the anterior chamber angle correlates with the lens-related parameters and the patterns of variation are needed.

## Data Availability

The datasets used and/or analysed during the current study are available from the corresponding author on reasonable request.
